# It Is All in the Blood: The Multifaceted Contribution of Circulating Progenitor Cells in Diabetic Complications

**DOI:** 10.1155/2012/742976

**Published:** 2012-04-03

**Authors:** Gian Paolo Fadini, Angelo Avogaro

**Affiliations:** ^1^Department of Medicine, University of Padua, 35100 Padua, Italy; ^2^Laboratory of Experimental Diabetology, Venetian Institute of Molecular Medicine (VIMM), 35100 Padua, Italy

## Abstract

Diabetes mellitus (DM) is a worldwide growing disease and represents a huge social and healthcare problem owing to the burden of its complications. Micro- and macrovascular diabetic complications arise from excess damage through well-known biochemical pathways. Interestingly, microangiopathy hits the bone marrow (BM) microenvironment with features similar to retinopathy, nephropathy and neuropathy. The BM represents a reservoir of progenitor cells for multiple lineages, not limited to the hematopoietic system and including endothelial cells, smooth muscle cells, cardiomyocytes, and osteogenic cells. All these multiple progenitor cell lineages are profoundly altered in the setting of diabetes in humans and animal models. Reduction of endothelial progenitor cells (EPCs) along with excess smooth muscle progenitor (SMP) and osteoprogenitor cells creates an imbalance that promote the development of micro- and macroangiopathy. Finally, an excess generation of BM-derived fusogenic cells has been found to contribute to diabetic complications in animal models. Taken together, a growing amount of literature attributes to circulating progenitor cells a multi-faceted role in the pathophysiology of DM, setting a novel scenario that puts BM and the blood at the centre of the stage.

## 1. The Burden of Diabetic Complications

 Diabetes mellitus (DM) has reached a worldwide growing epidemic diffusion. DM is associated with a significantly reduced quality of life and represents an important health and social problem. Most importantly, DM leads to severe complications in many organs and tissues through the induction of microangiopathy and macroangiopathy. Hyperglycemia-induced biochemical abnormalities, such as overactivation of PKC and MAPK, excess flux through the exosamine and polyol pathways, and production of advanced glycation end-products (AGEs), all stem from the high concentration of reactive oxygen species induced by the overflowing mitochondrial respiratory chain [1]. These damage pathways induce profound changes in vascular endothelial and smooth muscle cells and subsequent modifications of the extracellular matrix (ECM). DM increases 2-3-fold the risk of cardiovascular disease (CVD), owing to the widespread endothelial dysfunction, which is considered the first step in the atherogenetic process [2, 3]. Atherosclerotic vascular disease in DM is aggressive, multifocal, distal and develops earlier than in non-DM subjects. Importantly, other cardiovascular risk factors that typical associate with DM, such as hypertension, obesity and dyslipidemia, concur to the accelerated risk of CVD. Microvascular complications, including retinopathy, nephropathy, and neuropathy, develop as a consequence of structural and functional damage to the microcirculation of target organs. Typical morphological features include thickening of the basement membrane, loss of pericyte coverage, capillary rarefaction, excess deposition of stiff EMC components leading to reduced perfusion, atrophic changes, and fibrosis. All these morphological features are reflected by organ dysfunctions, including visual loss, impaired glomerular filtration or tubular resorption, reduced nerve conduction velocity. Importantly, organs that are less commonly recognized among the targets of diabetic microangiopathy are the myocardium, the lung, and the bone marrow (BM). 

## 2. The Plasticity of Circulating Progenitor Cells

 In the adult organism, the BM represents the privileged site of hematopoiesis and the reservoir of stem/progenitor cells. In the last decades, it has been recognized that the BM harbours small subsets of progenitor cells for multiple cell lineages, not limited to the hematopoietic system [4, 5]. These cells can leave the BM upon appropriate stimulation and migrate in peripheral organs through the bloodstream. The prevailing concept is that immature cells in the BM niche retain plasticity and can undergo a multilineage differentiation, recapitulating some developmental steps taking place in embryonic stem cells. The best known form of this phenomenon is endothelial differentiation of BM-derived cells, which gives rise to endothelial progenitor cells (EPCs) [6]. Cell-tracking experiments using BM chimeric mice expressing the green fluorescent protein (GFP) or other reporters found that BM-derived cells can repopulate several organs and tissues, differentiating into multiple phenotypes [7–9]. Similarly, the study of rare cases of human sex-mismatched transplantation allowed to follow the fate of BM-derived cells by looking at the signal of the sex chromosomes and showed repopulation of the myocardium, lungs, kidney, and gastrointestinal tract by donor-derived cells [10–13]. It should be noted that not all studies unequivocally confirm the ability of BM-derived cells to contribute to peripheral cellular phenotypes different from hematopoietic cells [14, 15]. This discrepancy may depend upon the use of different cell tracking methods, imaging techniques, and disease models.

## 3. Endothelial Progenitor Cells

EPCs are immature BM-derived cells which undergo differentiation into endothelial cells and participate in endothelial repair and neoangiogenesis [6]. EPCs are commonly defined and enumerated by flow cytometry based on the co-expression of stemness antigens (e.g., CD34 and/or CD133) and endothelial markers (e.g., KDR). EPCs can also be isolated from circulating mononuclear cells using disparate culture protocols yielding heterogeneous cell types (reviewed elsewhere [16]). Briefly, it should be taken into account that a net separation between EPCs and hematopoietic cells, either progenitor or myeloid lineage-committed cells, is not always possible. As a result, several cultured EPC phenotypes retain overlapping features with the hematopoietic system [17]. EPCs can be mobilized from the BM into the peripheral blood in response to many stimuli including tissue ischemia, cytokines, and growth factors [18]. Once in the bloodstream, EPCs home specifically to sites of vascular damage to repair the disrupted endothelium and to provide pro-angiogenic stimuli in an attempt to restore blood flow and counter shortage of oxygen and nutrients [19]. With these two seminal functions, it is easy to understand how EPCs act as an integrated component of the cardiovascular system, which is subjected to pathological changes and is also a target of therapy. Importantly, EPCs are profoundly altered in the setting of type 1 and type 2 DM [20]. Several antigenic EPC phenotypes (e.g., CD34+KDR+) are profoundly reduced in the blood of type 2 diabetic patients compared to controls, independently of concomitant risk factors [21]. Pauperization of EPCs in diabetes is thought to explain, at least in part, the high CVD risk associated with DM, as patients would be less able to repair the endothelial injury and to counter ischemia with neoangiogenesis. Indeed, there is a close negative correlation between the severity of vascular disease and the level of circulating EPCs in diabetic patients [22]. The reduction of EPCs may also intervene as a pathogenic factor in microangiopathy, as clinically significant correlations have been found in the setting of retinopathy, nephropathy, and wound healing [23–25]. Not only EPCs are reduced in the bloodstream of diabetic patients, but they also show functional defects, such as impaired adhesion, proliferation, and tubulogenesis [22, 26]. These data support the notion that an altered EPC biology in DM compromises the ability to counter the excess damage caused by hyperglycemia and the associated biochemical abnormalities [27]. Besides a pathophysiological role in diabetic complications, the level of circulating EPCs may also represent a biomarker of future risk, as progenitor cell counts independently predict the occurrence of adverse cardiovascular events in different cohorts of patients [28, 29].

## 4. Smooth Muscle Progenitor Cells

 Circulating SMPs were originally identified by studies in which mice were transplanted with genetically labelled BM and, after vascular injury, it was found that a quote of cells within the neointima coexpressed BM-tracing markers and alpha-SMA [7, 30, 31]. While these findings were not confirmed by other investigators [32, 33], data also accumulated on the possibility to isolate SMPs from peripheral blood mononuclear cells using different culture protocols (reviewed in [34]). The exact phenotype of SMPs is unclear and residual overlapping with the hematopoietic system (such as CD14 and/or CD45 expression) may occur as for EPCs. EPCs and SMPs may also share a common ancestor and cells may undergo shift from and back each phenotype in vitro and in vivo [35]. SMPs can be obtained from the CD34+ population and/or from the CX3CR1+ myeloid population [36, 37]. The existence of SMP has important implications for tissue engineering, as SM cells are necessary to create vascular grafts, but also holds negative implications for vascular disease, in which SM cells may play detrimental roles. In the setting of DM, SM cell function and phenotype are altered and some cells are shifted from a contracting phenotype to a secreting phenotype [38]. Nguyen et al. have reported that PBMC from diabetic patients as compared to controls, when cultured in conditions that foster SM cell growth, gives rise to a higher number of SM-like progenitor cells expressing both contractile and fibrogenous markers [39]. These findings were suggestive of the fact that circulating progenitors in DM are shifted from the generation of an endothelial phenotype to a SM phenotype. As it has been shown that BM-derived cells home to sites of glomerular damage [40], these cells may intervene in the setting of diabetic microvascular complications, contributing for instance in the progression of diabetic nephropathy. Consistently with these findings, In a mouse model of type 1 diabetes, Westerweel et al. found an accelerated generation of SMP from blood cells, probably driven by the TGF-beta signalling. In addition, GFP+ BM-derived cells coexpressing SM cell markers were recruited at sites of neointima formation after cuff arterial damage, which was increased in diabetic animals [41].

Pericyte progenitor cells (PPCs) represent a phenotype closely related to SMPs. They are typically defined by expression of pericyte markers, such as PGDFRbeta and/or NG2. PPCs can be isolated from mature blood vessels and show potent vasoregenerative potential [42], just as pericyte govern vascular stability. Circulating PPCs have been identified, although their origin is not clear [43]. We have reported that PPCs are increased in diabetic patients with microangiopathy after improvement of glucose control [44]. The clinical significance of this finding remains to be elucidated. Speculatively, increased vasoprotective PPCs may represent a beneficial effect of glucose control that translates into improved outcomes. Alternatively, the surge in PPC level may represent a consequence of microvascular lesion regression or even progression, as microangiopathy can occasionally worsen after rapid glucose control.

## 5. Cardiomyocyte Progenitor Cells

Early studies using GFP BM chimeric mice were able to detect an extensive repopulation of the infarcted myocardium by BM-derived cells, with initial evidence of transdifferentiation of homed cells into cardiomyocytes [8, 45]. In humans, a proof of concept of this biological phenomenon has been provided in sex mismatched heart transplants, showing high level of cardiac chimerism caused by the migration of primitive cells from the recipient to the grafted heart [10, 46, 47]. The phenotype and kinetics of BM-derived circulating cardiomyocyte progenitor cells (CPCs) have been subsequently investigated. For instance, Wojakowski et al. found that myocardial infarction induces the BM to release CD34/CXCR4+, CD34/CD117+, and c-Met+ progenitor cells, which express the cardiac genes GATA4, MEF2C, Nkx2.5/Csx [48]. The extent to which these cells engraft into the infarcted heart was not determined and the true quantitative contribution of BM-derived cells in myocardial remodelling after injury has been questioned [49, 50]. An important issue is that EPCs themselves have the potential to transdifferentiate into cardiomyocytes in vitro, when co-cultured with neonatal rat heart cells [51]. Interestingly, this phenomenon is impaired in patients with coronary artery disease and can be restored by statin therapy [52]. Whether cardiomyocyte differentiation of circulating progenitors is affected by diabetes remains to be established.

## 6. Osteoprogenitor Cells

 Ectopic calcification is a hallmark feature of diabetic vasculopathy [53]. Calcification can develop in the medial layer or in the neointima of atherosclerotic plaques. Medial calcified arteriosclerosis leads to arterial stiffening and raises blood pressure, while neointimal calcification can destabilize the plaque and lead to rupture or hemorrhage. The mechanisms driving vascular calcification are incompletely understood, ranging from ion imbalance, loss of inhibitors, and cellular alterations [54]. The type of vascular cells giving origin to calcifying cells is also unclear, with SM cells, pericytes, and advential progenitor cells possibly being involved. Recent data show that circulating calcifying cells (osteoprogenitor cells, OPCs) contribute to intraplaque calcification [55], while a role for BM-derived cells in medial calcification has not been substantiated so far [56]. OPCs may originate from the BM hematopoietic or mesenchymal compartments. It has been shown that CD34+ cells and EPCs can express bone-related proteins, such as osteocalcin (OC) and bone alkaline phosphatase (BAP) and develop a tendency to form calcified nodules in vitro and when using in vivo assays [57]. These osteogenic EPCs, or OPCs, are increased in patients with coronary artery disease, and data in humans suggest that they are recruited from the bloodstream to the diseased coronary arteries [57, 58]. Expression of OC on EPCs correlates with arterial stiffness in humans [59], lending support to the hypothesis that OPG participates in arterial calcification. In diabetic patients with coronary artery disease, CD34+ cells show a phenotypic shift from endothelial commitment to a procalcific phenotype, as evidenced from the excess OC expression over KDR [60]. In cultured monocytic EPCs, this phenomenon may be attributable to inflammatory stimuli, as it can be recapitulated by LPS [60]. In addition, we have recently identified a subpopulation of circulating monocytes expressing OC and BAP, called myeloid calcifying cells (MCCs), that are increased in the BM, peripheral blood, and atherosclerotic lesions of diabetic patients compared to controls [61]. MCCs represent one aspect of monocyte plasticity and a novel indicator of deranged monocyte biology in the setting of DM. Finally, OPCs may also derive from the BM mesenchymal compartment and can be mobilized into the bloodstream in response to bone fractures [62]. The complex epidemiologic and pathophysiologic relationships between bone and vascular disease suggest that OPCs may be involved in the regulation of the bone vascular axis [63], through yet unidentified mechanisms. In support of this, osteogenic EPCs appear to be increased also in osteoporotic women [64]. 

## 7. Proinsulin-Expressing Cells

A few years ago, while studying gene therapy in streptozotocin (STZ) diabetic mice, a group of investigators detected expression of the insulin gene in several organs and tissues outside the endocrine pancreas [65]. Then, they identified proinsulin- (PI-) expressing cells that appear in animals after induction of hyperglycemia. These cells derive from the BM, resemble cells of the monocyte/macrophage lineageand display a proinflammatory phenotype, as evidenced by the expression of TNF-alpha. When looking at the distribution of the PI-expressing BM-derived cells (PI-BMDCs) throughout the rodent organism, authors found these cells in multiple tissues and organs [66]. Importantly, PI-BMDCs appear to have enhanced fusogenic properties, at least in part mediated through the diabetes-specific PARP-1 pathway [67]. Fusion of PI-BMDCs with resident cells has been shown to contribute to diabetic complications [68]. For instance, fusion of hematopoietic cells with peripheral neurons impairs nerve function in a diabetic mouse model [69, 70]. Additionally, fusion of PI-BMDCs with renal tubule cells is believed to contribute to the development and/or progression of diabetic nephropathy, as the resulting polyploid cells are proinflammatory and interfere with normal tubule function [71]. The fusogenic properties of these cells are abolished when mice are transplanted with BM cells from PARP−/− donors [67]. Interestingly, an excess generation of these proinflammatory PI-expressing myeloid cells after development of diabetes may contribute to virtually all diabetic complications, by means of fusion with resident cells. This is an entirely new mechanism of action that link BM cells to distant target organs. Whether or not this mechanism is active also in humans needs to be addressed.

## 8. The Diabetic Bone Marrow

The profound alterations of all these circulating progenitors intuitively led investigators to hypothesize a BM defect associated with DM. In 2006, we first reported that BM mobilization of progenitor cells is impaired in diabetic animals compared to controls after stimulation by ischemia or exogenous mobilizing agents (G-CSF and SCF) [72]. The postischemic mobilization was defective in DM because ischemia was unable to upregulate the hypoxia sensing system HIF-1alpha and its downstream targets (such as SDF-1alpha), which signal the BM for the need of vasoregenerative progenitor cells, like EPCs. This pathway has been subsequently confirmed by others and defects of the HIF-1alpha pathway in DM have been better elucidated [73, 74]. On the other hand, to explain the impaired progenitor cell mobilization after direct BM stimulation, an intrinsic BM defect had to be postulated. Recently, Oikawa et al. have shown that DM induced BM microangiopathy with morphological features similar to other typical diabetic microvascular complications, including basement membrane thickening, capillary rarefaction and apoptosis [75]. As a functional consequence stem cell niche characteristics were altered, thus potentially affecting the BM response to mobilizing agents. Busik et al. have found that DM impairs autonomic bone marrow innervation, which is critical for G-CSF induced mobilization of stem/progenitor cells. This BM neuropathy, in turn, compromised the extent and timing of progenitor cell release, an event that preceded the development of distant vascular complications [76]. The early onset of bone marrow defect in the natural history of diabetes is also suggested by a study showing that CD34+ cells start to decline in prediabetes and show a first nadir in newly diagnosed type 2 DM [77]. More recently, Ferraro et al. showed that STZ diabetes in mice interrupts the dynamic anatomy of the BM stem cell niche suggesting a defect in the activation of the sympathetic nervous system with consequent impaired SDF-1alpha regulation. As a clinically relevant counterpart, they show in a retrospective case series that G-CSF stem cell mobilization in patients undergoing autologous transplantation is impaired in the presence of diabetes or hyperglycemia [78]. This issue is being explored in an ongoing prospective clinical trial in diabetic and non diabetic patients (NCT01102699), as a proof-of-concept for the so-called diabetic stem cell “mobilopathy” [79]. However, it is fascinating that complex niche dysfunction in DM may not only impair progenitor cell mobilization, but also affect differentiation of progenitor cells, with defective generation of EPCs and CPCs and excess production of SMPs, OPCs and PI-BMDCs that exert detrimental effects on diabetic complications [80].

## 9. Concluding Remarks

**Figure 1:**
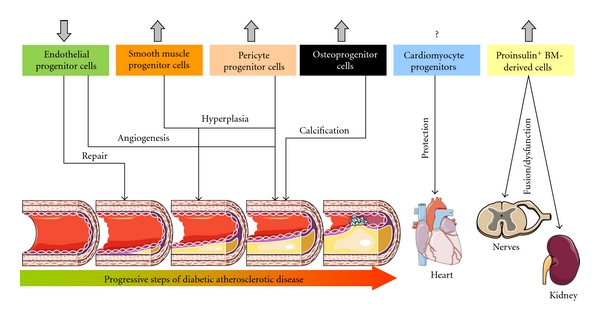
The multifaceted contribution of circulating progenitor cells in diabetic complications. Different lineage-committed progenitor cells are altered in the setting of diabetes and contribute to the development of diabetic complications. Grey arrows indicate the effects of diabetes of number and function of the various bone-marrow-derived cell subtypes.

The studies summarized so far currently attribute to circulating progenitors for multiple cell lineages important roles in the pathogenesis of diabetic complications. Progenitor cells typically originate from the BM and intrinsic BM alterations in DM begin to be characterized. Thus, the emerging scenario put the BM in the centre of a new pathophysiological model of diabetic complications, as a link between distant and disparate target organs (Figure 1). Importantly, stem cell failure is typically associated with aging and it is worth to note that, owing to the burden of complications, DM is considered a disease of accelerated aging [81].

At least some of the progenitor cell dysfunction found in DM are reversible [82–84]. For instance, glucose control with insulin therapy has been shown to increase EPCs [85] while normalization of glucose metabolism by islet transplantation in type 1 diabetes reversed EPC defects [86]. In addition, inhibition of DPP-4 with sitagliptin increased EPCs in 4 weeks in type 2 diabetic patients, possibly through an effect on SDF-1alpha [87]. Finally, the discovery of progenitor cell reduction in diabetes represents the rationale for devising cell-based therapeutic strategies [88], which show promising results for both coronary and peripheral vascular disease [89, 90].

Despite these data, several aspects of progenitor cells biology in DM still need to be extensively investigated. Among all, the monocyte plasticity and its deranged polarization [91], which is thought to account for unbalanced EPC, SMP, MCC and PI-BMDC generation, deserve a special attention.
